# Data set of balance of Russia's regional economy in 2005–2024 based on the methodology of calculation of “underdevelopment whirlpools”

**DOI:** 10.1016/j.dib.2020.105821

**Published:** 2020-06-10

**Authors:** Elena G. Popkova, Ksenia V. Ekimova, Bruno S. Sergi

**Affiliations:** a MGIMO University, Russia; b State University of Management, Russia; c Harvard University, USA; d University of Messina, Italy

**Keywords:** Data set, Balance, Regional economy, Russia, “Underdevelopment whirlpool”

## Abstract

The article shows the possibilities of Data Set “Interactive Statistics and Intelligent Analytics of the Balanced State of the Regional Economy of Russia in Terms of Big Data and Blockchain – 2020”. For creation of the data set, we formed time rows of the values of the selected indicators, which characterize the balance of Russia's regional economy. The indicators are systematized and classified into two categories. The first category includes the indicators of the level of socio-economic development: GDP per capita and “underdevelopment whirlpools”, balanced financial result of companies’ activities, population's employment level, and balance of the regional budget, calculated by finding the difference between revenues and expenditures. The second category includes the indicators of potential of socio-economic development: investments into fixed capital per capita, share of innovations-active organizations, share of innovative products, and digitization level. The data are collected in a table, based on which – with the help of programming and web-design – a data set is created – an interactive platform for working with data. The data set is available in Russian and English at the official web-site of the Institute of Scientific Communications [12]. The data set unifies and transfers into the digital form the data on the level and potential of Russia's regions’ potential of socio-economic development. The data set allows for flexible setting of data for any research. The data set allows – based on the statistics of a region's socio-economic development – determining its position in the Russian national ranking, category by the “TRMS” matrix, calculating the integral index for each region, and comparing its values. Using the proprietary methodology of calculation of “underdevelopment whirlpools” of Prof. Popkova, the data set reflects the balance of Russia's regional economy in 2005–2024. The data set allows for automatic visualization of data by creating a blockchain polygon of region's socio-economic development, which includes the chronogram of the region's development and the process of region's pulling into an “underdevelopment whirlpool”. The data set's data are presented in the form of an interactive map of Russia's regional economy. Map's color shows categories that are assigned to regions and the borders of regions, as well as information on each region's position in the 2020 rating. The data set allows for large-scale studies of Russia's regional economy with application of technologies of Big Data processing, machine learning, and intellectual analytics.

Specifications table

**Table:** 

Subject	Economics and econometrics
Specific subject area	Balance of regional economy
Type of data	Table, image, chart, graph, MS excel file, dataset
How data were acquired	For creation of the data set, we formed time rows of the values of the selected indicators, which characterize the balance of Russia's regional economy. The indicators are systematized and classified into two categories. The first category includes the indicators of the level of socio-economic development: GDP per capita and “underdevelopment whirlpools”, balanced financial result of companies’ activities, level of population's employment, and balance of regional budget, calculated by finding the difference between revenues and expenditures. The second category includes the indicators of potential of socio-economic development: investments into fixed capital per capita, share of innovations-active organizations, share of innovative products, and level of digitization. For calculation of “underdevelopment whirlpools” we used the data for GDP per capita for 2005–2018, and for other indicators – the data for two recent years – 2017–2018 for most indicators, and 2018–2019 for the level of digitization. Based on the calculated growth rate of the indicators’ values and based on expert analysis of strategies perspectives of development of Russia's regional economy, we compile a forecast with “other conditions being equal” for all indicators for the period until 2024.
Data format	Raw, analyzed, filtered
Parameters for data collection	The main parameter for data collection is their compatibility. S strict list of Russia's regions was determined – without federal cities (they are considered in the data for regions on which territory they are located).
Description of data collection	Based on the data for GDP per capita in Russia's regions and the proprietary methodology of calculation of “underdevelopment whirlpools” of Prof. Popkova, we calculated ”underdevelopment whirlpools” for all Russia's regions. Also, a methodology of compilation of a ranking of Russia's region was developed – it contains a formula for calculating the integral index and the hierarchical approach to classification of regions by the level and rate of their socio-economic development – “TRMS” matrix.
Data source location	The source of the data is the annual statistical collection “Regions of Russia: socio-economic indicators”, issued by the Federal State Statistics Service of the Russian Federation (Rosstat) [10] and the report “Digital Russia”, issued by the center for financial innovations and non-cash economy of Moscow School of Management “Skolkovo” [11] (Moscow, Russia).
Data accessibility	The data are gathered in a table, based on which – with the help of programming and web-design – a data set is created – an interactive platform. It is available in Russian and English at the official web-site of the Institute of Scientific Communications. In a public repository: Repository name: Data Set “Interactive Statistics and Intelligent Analytics of the Balanced State of the Regional Economy of Russia in Terms of Big Data and Blockchain – 2020″ Direct URL to data: https://www.archilab.online/en/data/date-set-on-the-regional-economy

## Value of the data

• The data set unifies and transfers into the digital form the data on the level and potential of Russia's regions’ socio-economic development, which is useful for scientific research on the topic of Russia's regional economy and for international comparisons of regional economies of different countries. The data set contains data selection for a long time period – 2005–2024. The data set allows compensating for the lack of official statistics on Russia's regional economy, caused by delayed provision of data. For example, in early 2020 the data only for 2018 are available – this problem is solved with the help of the author's forecasts.
• The data set will be useful for scholars who study Russia's regional economy. It allows selecting the necessary data and receiving the ready array of data in a Microsoft Excel table. This allows for large-scale studies of Russia's regional economy with application of the technologies of Big Data processing, machine learning, and intellectual analytics. The process of data selection is very simple – regions are grouped by federal districts, which allows using the data set by Russian and foreign researchers, who do not know the economic geography of Russia's regions very well.
• The data set allows for quick formation of data selections for studies on the topic of Russia's regional economy and convenient ranking of the data: one click can place the data in the countries’ alphabetical order or by increase or decrease of any indicator's values and group the data by the indicators or years. The data set allows for flexible setting of the data for any type of research. The forecast values of the indicators, presented in the data set, allow for studies based on the unified data, instead of compilation of a forecast in each study.
• Based on the statistics of region's socio-economic development, the data set allows determining its position in the Russia's national ranking, calculating the integral index for each region, and comparing its values. The order of recount of the regions’ categories in the “TRMS” matrix reflects the logic of their evolution. That's why the category assigned to a region shows its current position and perspectives of its changes in future – therefore, the data set is especially useful for forecasting during scientific research.
• Data set allows for automatic visualization of data through creation of a blockchain polygon of region's socio-economic development, which includes a chronogram of the region's development (dynamics of the depth of “underdevelopment whirlpool” and its position as relating to zero) and the process of region's pulling into an “underdevelopment whirlpool”.
• For illustration purposes, the data of the data set are presented in the form of an interactive map of Russia's regional economy. Color denotes the categories assigned to regions; it is possible to clearly see the borders of the regions, with information on position of each region in the 2020 ranking.

## 1 Data description

It is necessary to select the necessary data from the data set. Initially, one should select the data units, which could be regions and federal districts (aggregated units of data, which unify a lot of regions). In this paper, we consider the usage of data set by the example of federal districts.

**Fig. 1:**
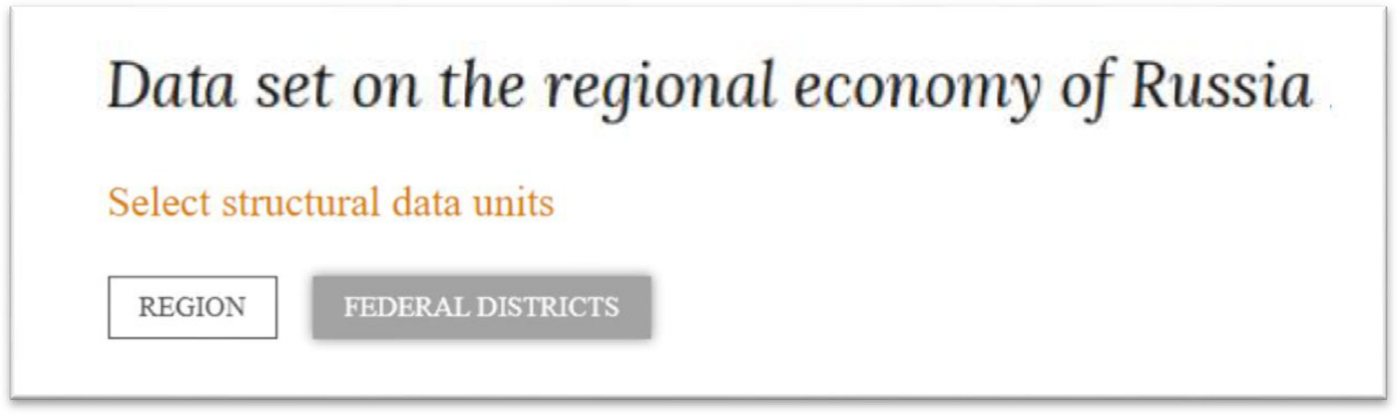
Screenshot of the dialog window of the data set with the user during selection of structural units of data – federal districts are selected.

Source: [12].

A screenshot of the dialog window of the data set with the user during selection of structural units is shown in Fig. 1.

**Fig. 2:**
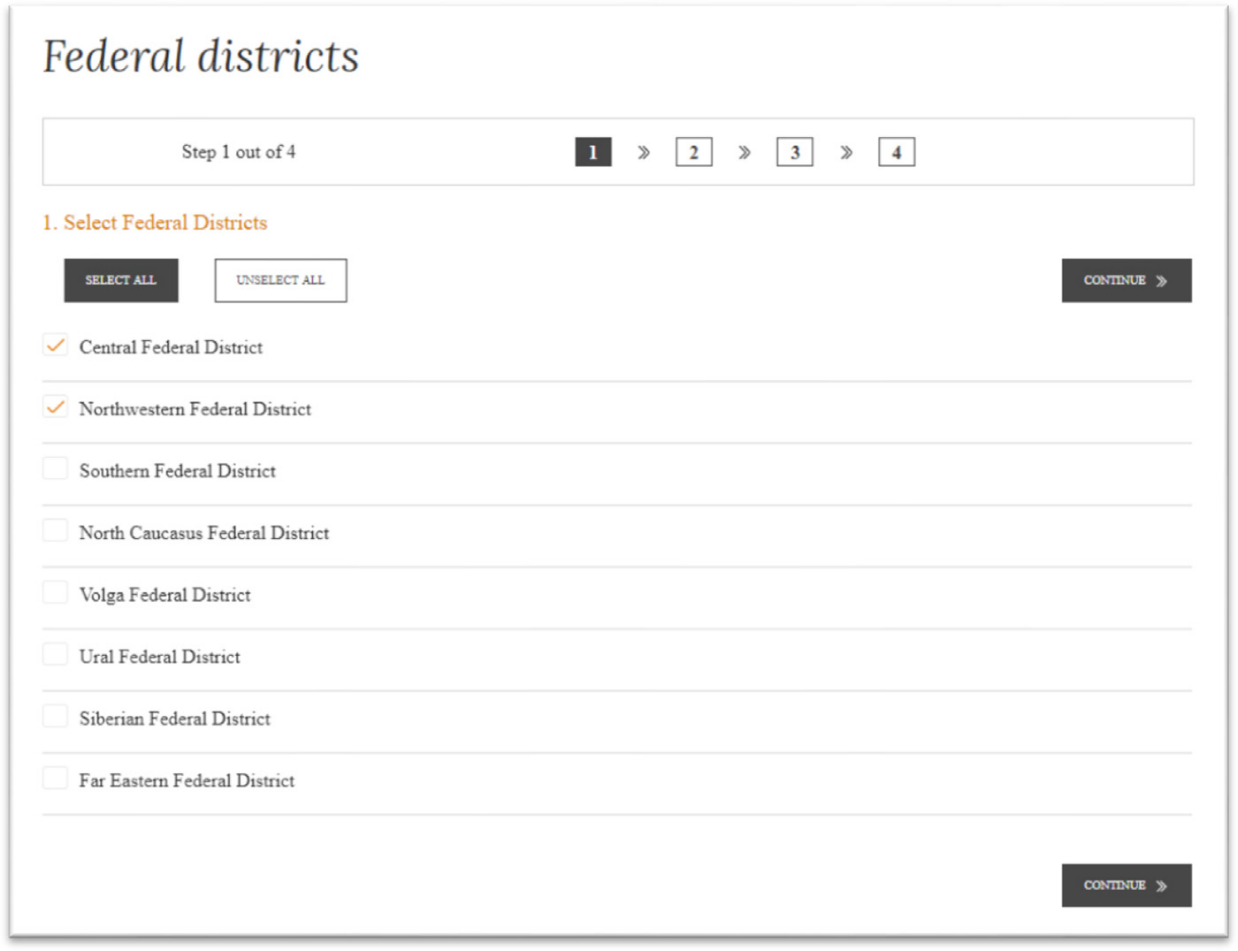
Screenshot of the dialog window of the data set with the user at Step 1 during selection of federal districts.

Source: [12].

After selection of structural units, the user goes to Step 1, to selection of federal districts (Fig. 2).

**Fig. 3:**
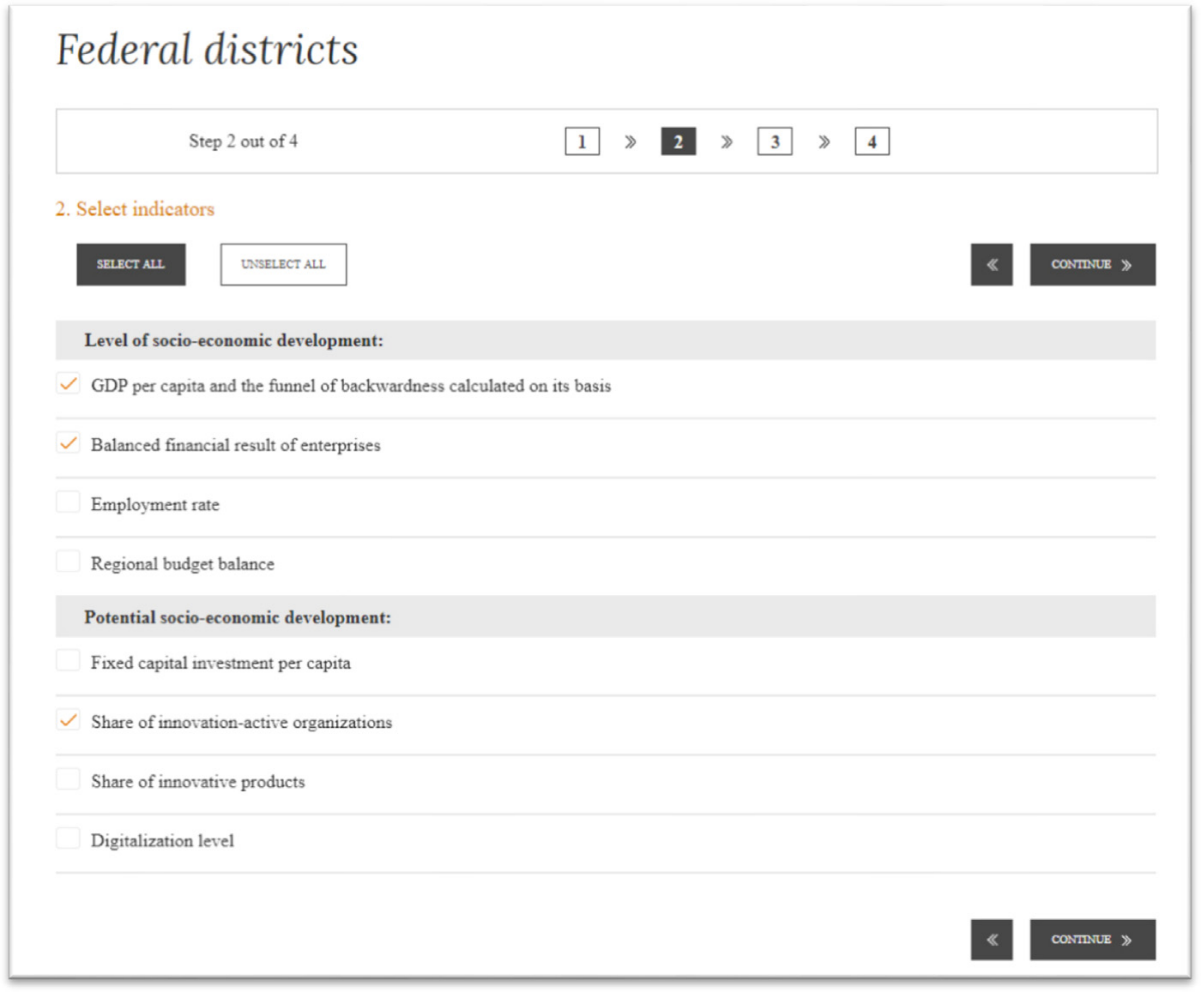
Screenshot of the dialog window of the data set with the user at Step 2 during selection of indicators.

Source: [12].

The user can “select all”, “unselect all”, and select certain federal districts manually by ticking them. Expanded options are available for regions: it is possible to select regions of a certain category or a specific federal district. Regions are grouped by federal districts, for maximum simplification of the geographical aspect of data selection for the user. Then, the user goes to Step 2, to select the indicators from the list (Fig. 3).

**Fig. 4:**
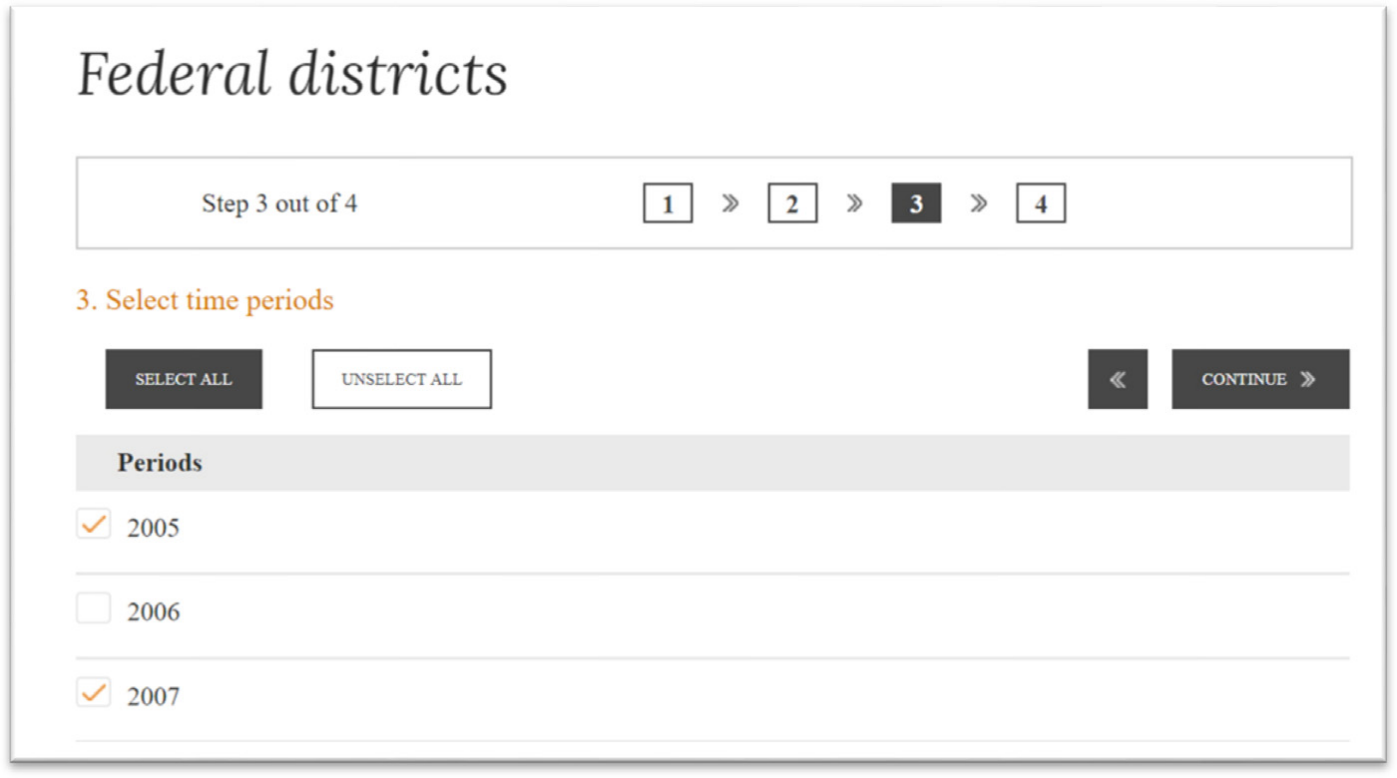
Screenshot of the dialog window of the data set with the user at Step 3 during selection of time periods (calendar years).

Source: [12].

Here the user can select all indicator or only specific indicators. Then, at Step 3 the user selects time periods (calendar year) for which the user requires the data (Fig. 4).

**Fig. 5:**
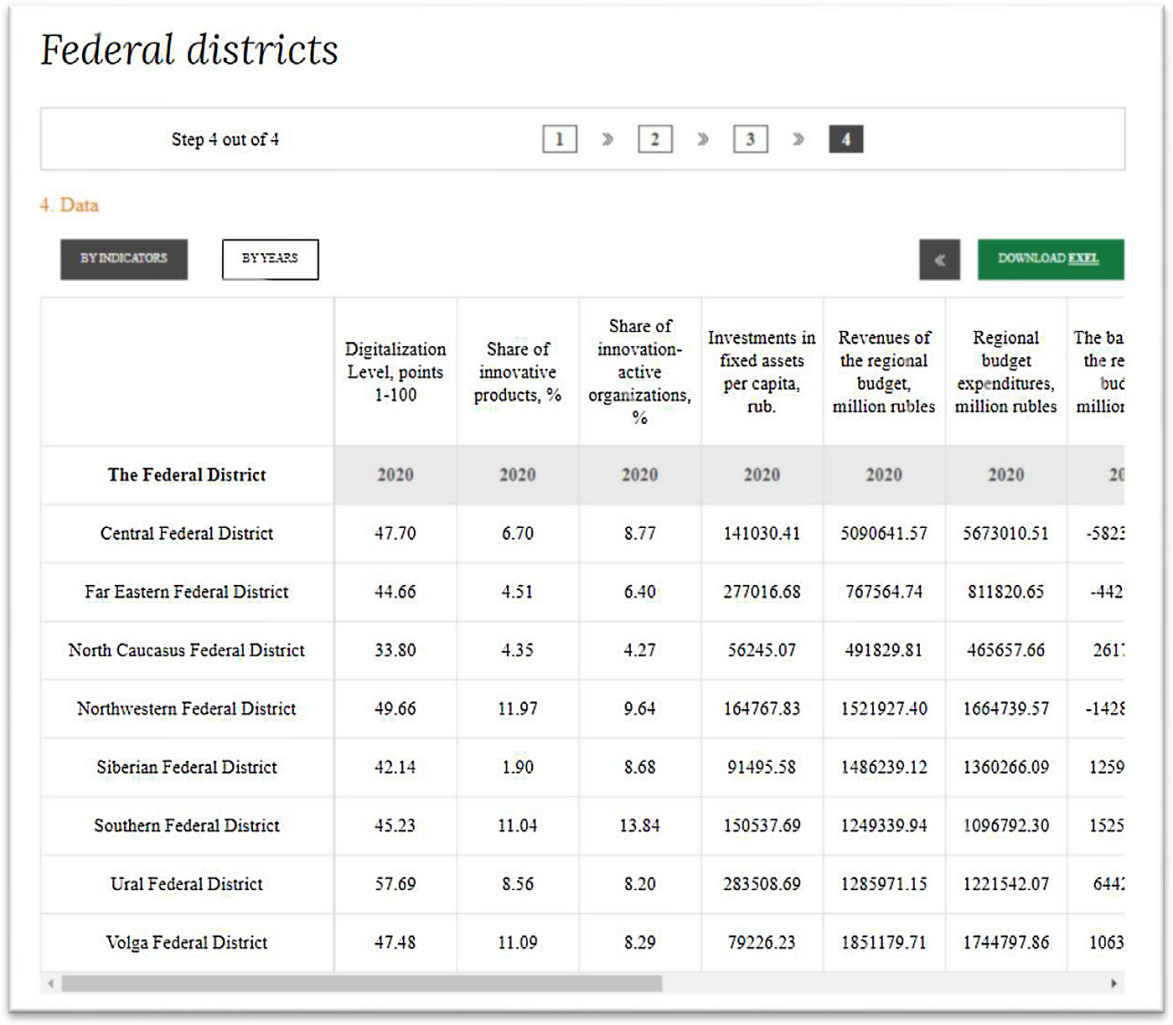
Screenshot of the table of data, provided to user by the data set, for 2020.

Source: [12].

Fig. 4 shows the start of the dialog window – in reality, the list of years is very long, covering 2005–2024. The data for 2005–2016 are available only for GDP per capita and “underdevelopment whirlpools”, and the data for 2020–2024 are forecast data. After selection of years, a table of data is formed (Fig. 5).

The forecast period is 2020–2024 because the current stage of development of the Russian economic is oriented at the period until 2024. This was stated in the 2018 paper “Russia-2024: Strategy of socio-economic development” by the Prime-Minister of the Russian Federation D.A. Medvedev [1b]. The Decree of the President of the Russian Federation dated May 7, 2018 No. 204 “Regarding the national goals and strategic tasks of development of the Russian Federation until 2024” is also oriented at the period until 2024. The program of modernization of the Russian economy “Digital economy of Russia 2024” is oriented at the period until 2024.

Therefore, forecasts of development of Russia and its regional economy should be oriented at the period until 2024, in order to cover the current stage of this development and to guarantee maximum precision and correctness of the forecasts, for after 2024 the conditions of the Russian economy's functioning could change, which will not allow for implementation of the forecast. Thus, forecasting for a larger period (after 2024) is senseless.

As the conditions of the Russian economy's development until 2024 are rather stable and are controlled by the government – even during the 2020 global crisis – the federal government provides support for regions and implements the principle of budget federalism – forecasting in the data set is performed by the principle “all other conditions being equal”. The forecasting methodology is based on a two-stage algorithm. At the first stage, annual growth rate of each indicator of the data set is determined. At the second stage, a product of the indicators’ values in each studied year and their growth rate is calculated. For example, the 2020 forecast value is a product of the 2019 factual value and the 2019 growth rate of the indictor (as compared to 2018), etc. The forecast is to form the basis for scientific studies of the Russian regional economy.

Fig. 5 shows that user can group the data in any way: by the indicators or by years. The data could be listed by countries (alphabetically – click on “federal district”), or by increase or decrease of their values by any indicator (click on the indicator). “Download EXEL” starts downloading the final table of data on the user's PC. Microsoft Excel provides a table of data that are grouped by years and the indicators. The full selection of statistical data, which are available in the data set, for 2019–2020 is shown in Table 1.

**Table 1:** Statistics of socio-economic development of Russia's regions in 2019–2020: selection of data from the data set.

The Federal District (FD)	Digitalization level, points 1–100	Share of innovative products,%	Share of innovation-active organizations,%	Investments in fixed assets per capita, RUB	Revenues of the regional budget, RUB million	Regional budget expenditures, RUB million	The balance of the regional budget, RUB million	Employment rate,%	Balanced financial result of enterprises, RUB million	Backward-funnels: GDP per capita RUB	Corresponding year of reference	Depth of the funnel of backwardness, years	The rate of pulling into the "funnel of retardation", years for 1 year
**2019**													
Central FD	47.23	6.76	9.13	128209.46	4586163.58	4933052.61	−346889.03	69.00	1406943.89	762045.00	2022	−3	0
Far Eastern	44.22	4.10	6.40	247336.32	759965.09	795902.60	−35937.50	67.77	72733.16	786716.00	2023	−4	0
North FD Caucasus FD	33.46	4.78	3.88	54606.86	459654.03	443483.48	16170.54	58.30	15505.32	224483.00	2008	11	0
Northwestern FD	49.17	9.65	9.27	153988.63	1409192.03	1513399.61	−104207.58	68.10	1610539.77	825316.00	2023	−4	0
Siberian FD	41.73	2.21	8.19	87138.64	1376147.33	1295491.52	80655.82	60.89	1285234.16	466818.00	2016	3	0
Southern FD	44.78	10.32	11.73	124411.32	1105610.57	1006231.47	99379.10	63.00	276780.24	368470.00	2013	6	0
Ural FD	57.12	7.25	8.20	264961.39	1201842.20	1163373.40	38468.80	65.40	1146590.70	931961.00	2024	−5	1
Volga FD	47.01	11.79	8.55	80026.49	1746395.95	1677690.25	68705.70	63.67	23790.40	424872.00	2014	5	1
**2020**													
Central FD	47.70	6.70	8.77	141030.41	5090641.57	5673010.51	−582368.93	69.00	872305.21	817889.85	2023	−3	0
Far Eastern	44.66	4.51	6.40	277016.68	767564.74	811820.65	−44255.90	68.45	37093.91	857748.41	2024	−4	0
North FD Caucasus FD	33.80	4.35	4.27	56245.07	491829.81	465657.66	26172.15	58.30	12559.31	239665.19	2008	12	1
Northwestern FD	49.66	11.97	9.64	164767.83	1521927.40	1664739.57	−142812.18	68.10	1610539.77	937891.80	2024	−4	0
Siberian FD	42.14	1.90	8.68	91495.58	1486239.12	1360266.09	125973.03	60.28	1310938.84	504810.64	2017	3	0
Southern FD	45.23	11.04	13.84	150537.69	1249339.94	1096792.30	152547.64	63.00	213120.78	395226.34	2014	6	0
Ural FD	57.69	8.56	8.20	283508.69	1285971.15	1221542.07	64429.09	65.40	1031931.63	998017.74	2024	−4	1
Volga FD	47.48	11.09	8.29	79226.23	1851179.71	1744797.86	106381.85	63.03	6185.50	453283.04	2015	5	0

**Fig. 6:**
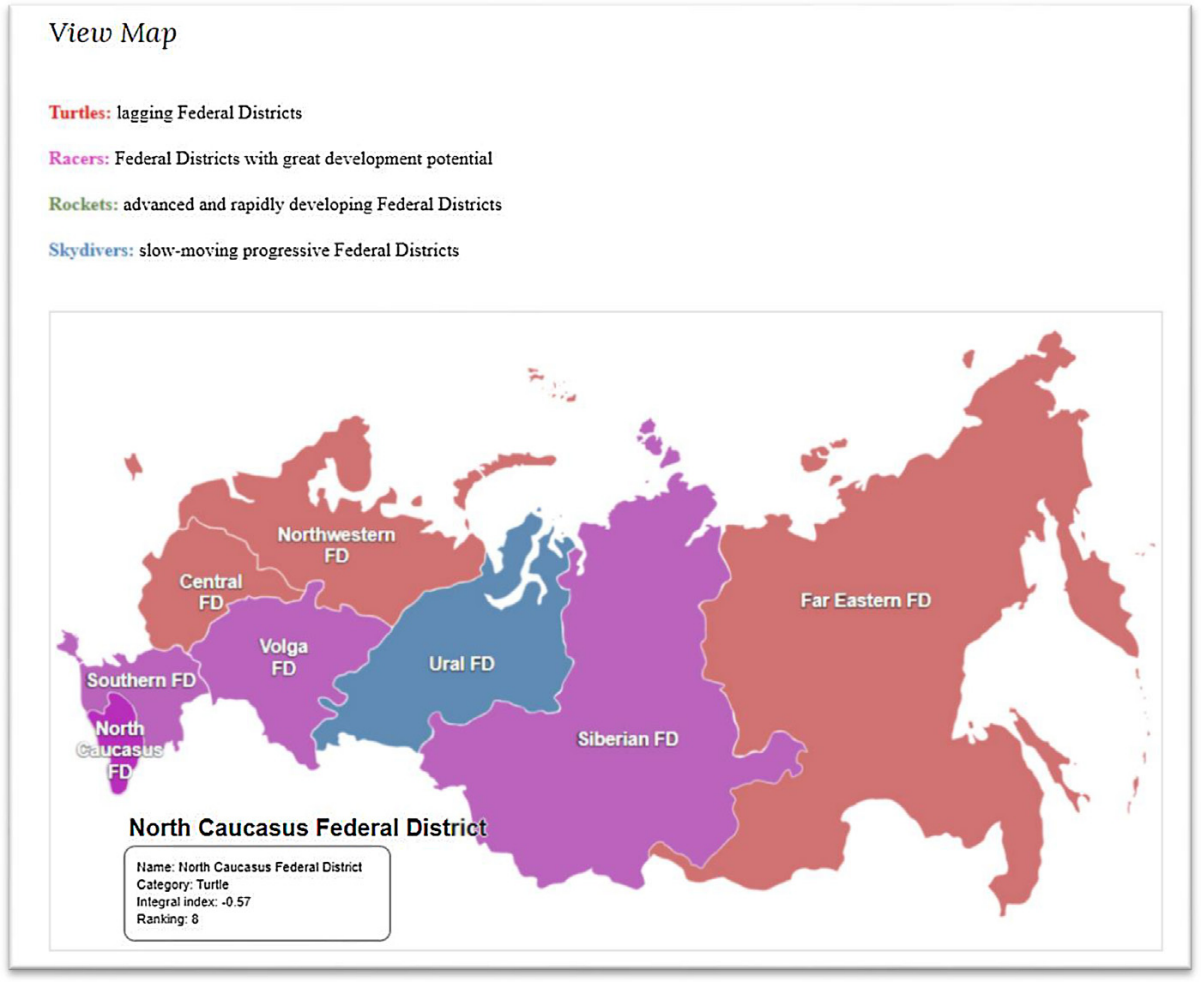
Screenshot of the interactive map of Russia's federal districts for 2020 from the data set.

Source: [12].

For illustration purposes, the data for 2020 are shown in the form of maps – an interactive map of regions and federal districts of Russia is presented (Fig. 6).

As shown in Fig. 6, color in the map shows the level of socio-economic development of each federal district in 2020, according to the distinguished categories. Red – “turtles”, purple – “racers”, green – “rockets”, and blue – “parachutists”. User has to place the cursor on a federal district for seeing the information on the level of its socio-economic development: category, integral index, and position in the national ranking for 2020.

**Fig. 7:**
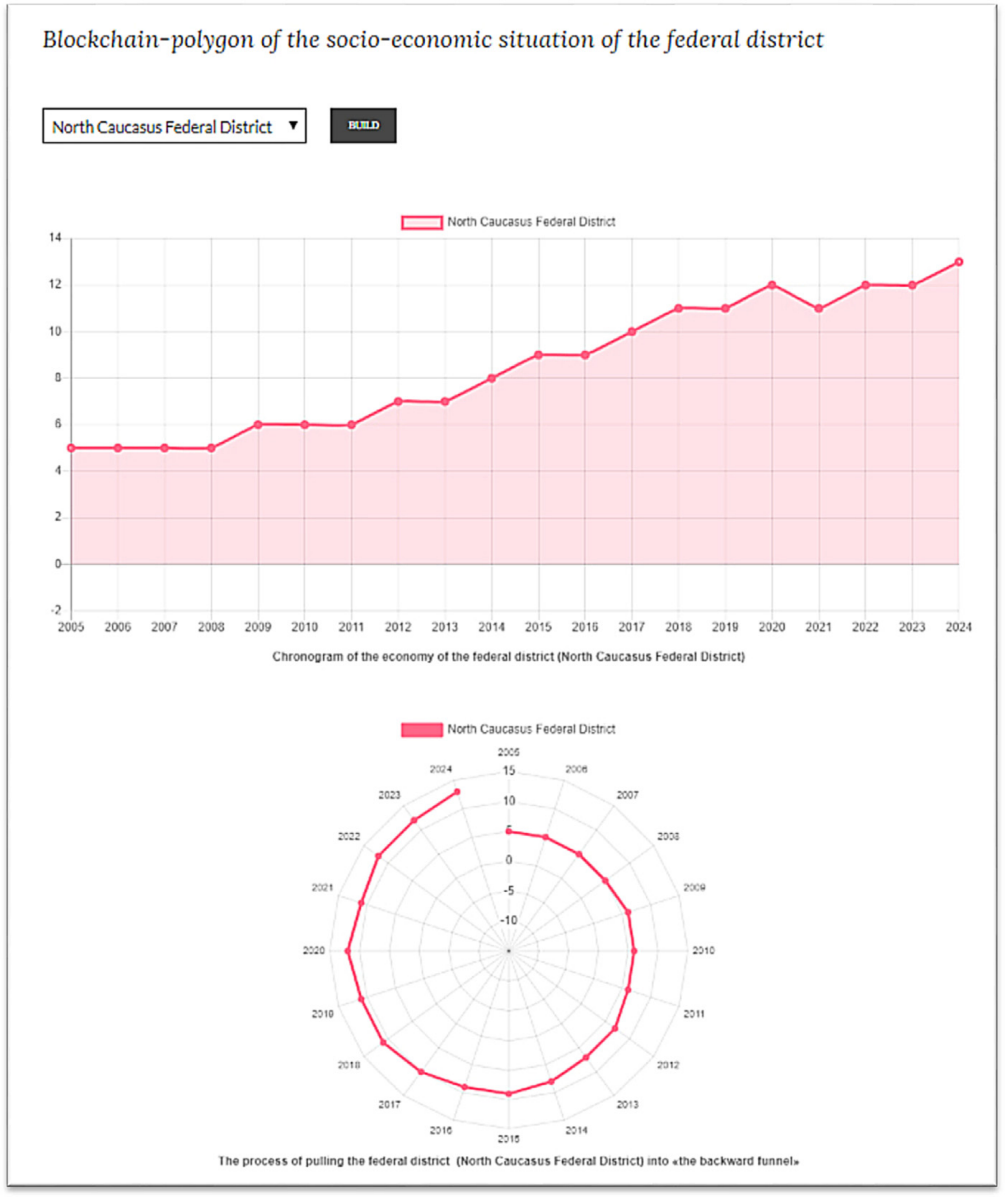
Screenshot of a blockchain polygon of the level of socio-economic development of a federal district, built automatically by the data set.

Source: [12].

User can automatically build a blockchain polygon of the level of socio-economic development of a federal district (Fig. 7).

**Fig. 8:**
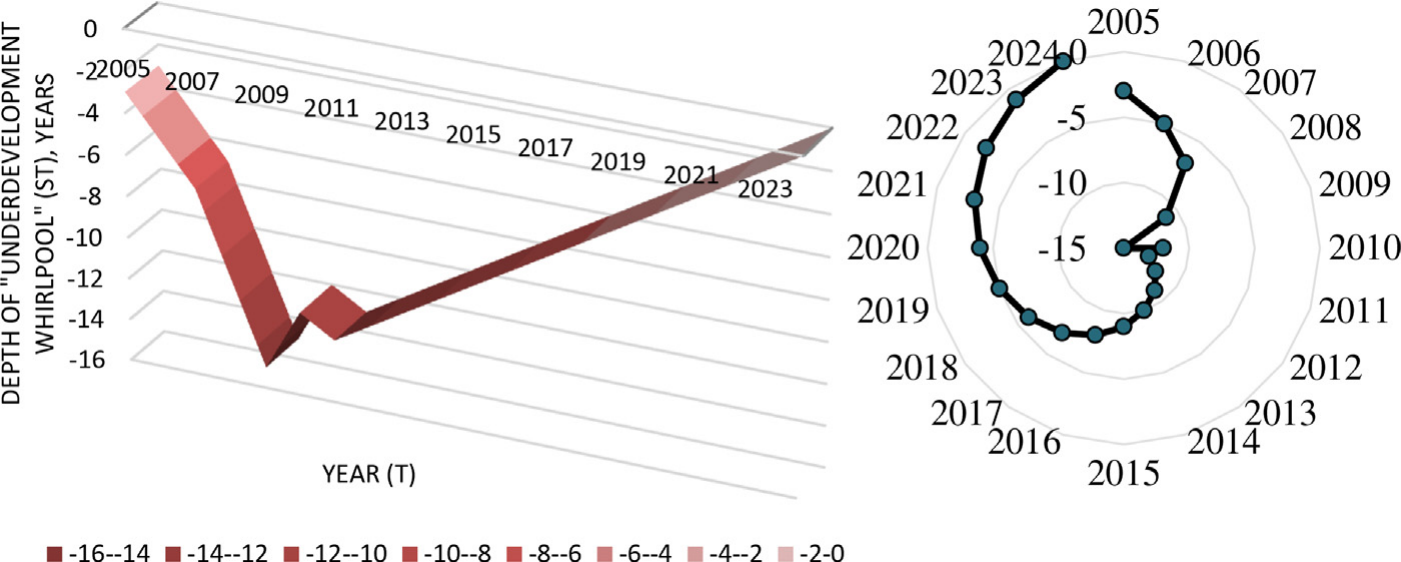
Three-dimensional chronogram (left) and polygon of the depth of “underdevelopment whirlpool” in Chukotka Autonomous Okrug in Russia in 2005–2024.

Source: built by the author in Microsoft Excel based on [12].

Though the possibilities of creation of graphs and diagrams in the data set are limited by the set of available graphic objects, it is possible – based on the data provided by the data set – to build three-dimensional graphs and various diagrams in any software that supports data export from Microsoft Excel (Fig. 8).

At the final stage of usage of the data set, user sees a rating of regions and rating of federal districts of Russia by the level of socio-economic development in 2020 (Table 2).

**Table 2:** Rating of Russia's federal districts by the level of socio-economic development in 2020, in the data set.

Federal districts of the Russian Federation	Category	Integrated rating for 2020, points	Place by the end of 2020
Southern Federal District	Turtle	3.36	1
Siberian Federal District	Turtle	3.35	2
Ural federal district	Rocket	2.94	3
Volga Federal District	Turtle	2.22	4
Northwestern Federal District	Racer	1.68	5
Central Federal District	Racer	1.26	6
Far Eastern Federal District	Racer	1.11	7
North Caucasus Federal District	Turtle	−0.57	8

For users’ convenience, it is possible to rank regions in alphabetical order, by categories, and by decrease or increase of the values of the integral index and position in rating.

## 2 Experimental design, materials, and methods

The methodology that is used during creation of a data set is based on calculation of “underdevelopment whirlpools”. The proprietary methodology of Prof. Popkova, described and well proven in the following publications (but not limited to them) is used for the calculation of the depth of “underdevelopment whirlpools” in the regions and federal districts of the Russian Federation [[1], [2], [3], [4], [5]].

It should be noted that there are a lot of studies devoted to the Russian regional economy and the methodology of evaluation of regional economy's balance [[6], [7], [8], [9]]. However, the existing literary sources focus on the current level of socio-economic development of region, but dynamics and perspectives of its development are neglected. The methodology of calculation of “underdevelopment whirlpools” allows overcoming this drawback and characterizing regions’ socio-economic development – in statics and dynamics.

The “underdevelopment whirlpool” be understood as the degree of lagging of the economic system from the reference system in terms of the level of GDP per capita – in this particular case, of a region or federal district from the average level of GDP per capita in the Russian Federation. The “underdevelopment whirlpool” has a static measure – the depth in the specified year – and dynamic indicator – the rate of pulling into the “underdevelopment whirlpool” for 1 year.

The depth of the “underdevelopment whirlpool” is determined as follows. Firstly, the value of GDP per capita based on current (market) prices for a particular year is taken from the official statistics. Then a year is determined in which the closest value of GDP per capita for the entire Russian Federation (reference value) could be observed. After that, the depth of the “underdevelopment whirlpool” is determined as the difference between the current year and the relevant year. Then the rate of pulling in the “underdevelopment whirlpool” is determined as the difference between the depth of the “underdevelopment whirlpool” in the current year and its depth in the previous year.

The values of GDP per capita in the Russian Federation are taken as reference values. Since official statistics present data for this indicator only for the period from 1995 till 2016 these data are supplemented for the computational analysis with author's estimation for the period from 1965 till 1994, as well as author's forecast for 2017–2024.

According to the proprietary methodology, “underdevelopment whirlpools” are calculated in the following way. The value of GDP per capita – gross value added per capita at current (market) prices in the current year (e.g., 2020) – is taken. Then, the corresponding year of reference is determined – the year in which the closest value of GDP per capita was observed in the Russian Federation (standard, from Table 3). After this, the depth of the “underdevelopment whirlpool” is calculated, in years – the difference between the current year and the corresponding year. Then, the rate of pulling into the "underdevelopment whirlpool" is calculated, years for 1 year – the difference between the depths of the "underdevelopment whirlpool" in the current year and its depth last year.

**Table 3:** Inter-temporal changes in GDP per capita in the Russian Federation in 1965–2024.

Year	GDP per capita in the Russian Federation, RUB	Year	GDP per capita in the Russian Federation, RUB	Year	GDP per capita in the Russian Federation, RUB	Year	GDP per capita in the Russian Federation, RUB
1965^a^	10.6	1980^a^	304.9	1995	13230.0	2010	263829.0
1966^a^	13.3	1981^a^	381.4	1996	15212.3	2011	317515.0
1967^a^	16.6	1982^a^	477.1	1997	16590.8	2012	348642.0
1968^a^	20.8	1983^a^	596.7	1998	28492.1	2013	377006.0
1969^a^	26.0	1984^a^	746.4	1999	39532.3	2014	405148.0
1970^a^	32.5	1985^a^	933.6	2000	49474.8	2015	449098.0
1971^a^	40.7	1986^a^	1167.7	2001	60611.4	2016	472162.0
1972^a^	50.9	1987^a^	1460.6	2002	74884.9	2017^b^	509066.0
1973^a^	63.7	1988^a^	1826.9	2003	97864.8	2018^b^	548855.0
1974^a^	79.6	1989^a^	2285.1	2004	126014.0	2019^b^	591754.0
1975^a^	99.6	1990^a^	2858.1	2005	157854.0	2020^b^	638005.0
1976^a^	124.6	1991^a^	3575.0	2006	196770.0	2021^b^	687872.0
1977^a^	155.8	1992^a^	4471.6	2007	241767.0	2022^b^	741636.0
1978^a^	194.9	1993^a^	5593.0	2008	226008.0	2023^b^	799603.0
1979^a^	243.8	1994^a^	6995.8	2009	226008.0	2024^b^	862100.0

a Author's estimation;.

b Author's forecast “all other factors held equal”. Source: [12].

**Fig. 9:**
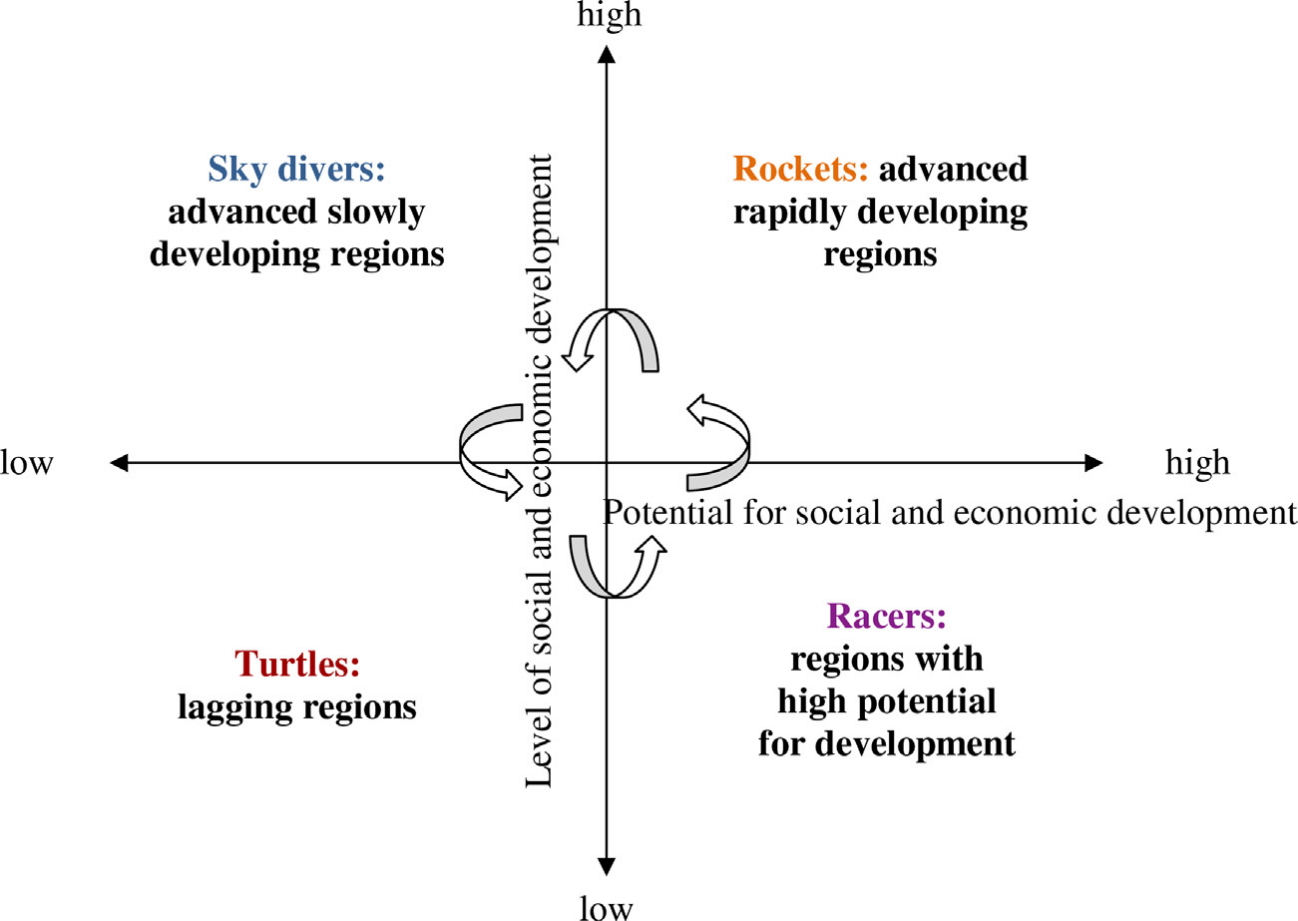
“TRMS” matrix of social and economic development of regions

Source: [12].

Also, the following methodology for drawing up the rating of Russian regions is used. When drawing up a rating of Russian regions, the ISC is guided by the two criteria – level and potential for social and economic development – and the logic shown below (Fig. 9).

As can be seen from Fig. 9, ISC distinguishes between four categories of regions according to level and potential of their social and economic development:

1. *Turtles:* lagging regions. They are shown in the bottom left sector of the matrix. For these regions, the depth of the “underdevelopment whirlpool” is below zero, and the arithmetical mean of indicators of the potential for social and economic development (the ratio to the average Russian value of fixed investment per capita, the share of innovatively active organizations, the share of innovative products and digitization level) is less than 1; in addition, the following characteristics can be observed (but not necessarily simultaneously): the balanced financial result of the business is less than 0 (+), the ratio of occupational level to the average level in Russia is less than 1, and the budget balance of the constituent entity of the Federation is less than 0 (+).
2. *Racers:* regions with high potential for development. They are shown in the bottom right sector of the matrix. They are characterized by the depth of the “underdevelopment whirlpool” which is less than or equal to 0 (there is no “underdevelopment whirlpool”), but the following conditions are not observed simultaneously at that: the balanced financial result of the business is greater than or equal to 0 (+), the ratio of occupational level to the average level in Russia is greater than 1, and the budget balance of the constituent entity of the Federation is greater than or equal to 0 (+), and only some of these conditions are observed, whereas the arithmetical mean of indicators of the potential for social and economic development (the ratio to the average Russian value of fixed investment per capita, the share of innovatively active organizations, the share of innovative products and digitization level) is less than 1.
3. *Missiles:* advanced rapidly developing regions. They are shown in the right sector of the matrix. The following conditions are simultaneously observed for these regions: the depth of the “underdevelopment whirlpool” is less than or equal to 0 (there is no “underdevelopment whirlpool”), the arithmetical mean of indicators of the potential for social and economic development (the ratio to the average Russian value of fixed investment per capita, the share of innovatively active organizations, the share of innovative products and digitization level) is greater than 1, and almost all of the following conditions are observed: the balanced financial result of the business is greater than or equal to 0 (+), the ratio of occupational level to the average level in Russia is greater than 1, and the budget balance of the constituent entity of the Federation is greater than or equal to 0 (+).
4. *Sky divers:* advanced slowly developing regions. They are shown in the upper left sector of the matrix. For these regions, the depth of the “underdevelopment whirlpool” is greater than zero, the balanced financial result of the business is greater than or equal to 0 (+), the ratio of occupational level to the average level in Russia is greater than 1, and the budget balance of the constituent entity of the Federation is greater than or equal to 0 (+), whereas the arithmetical mean of indicators of the potential for social and economic development (the ratio to the average Russian value of fixed investment per capita, the share of innovatively active organizations, the share of innovative products and digitization level) is greater than 1.

The order in which categories of regions are listed is not random – it is reflective of the logic of their evolution. At first, the region is lagging (T), then it accumulates potential (R), after which it unlocks this potential (M), exhausts it and is lagging in development (S), and eventually it may become lagging again. This is a vivid example of cyclic development of regions.

The rating is drawn up for each individual region and federal districts of the Russian Federation. The higher is the value of the integral index, the higher is the rating position of the constituent entity of the Federation. The *integral index* is determined as follows:

(1) II=(LD+PD)/2,

where II is the integral index;

LD is the arithmetical mean of the depth of the “underdevelopment whirlpool” reversed in sign (multiplied by −1), the ratio to the average Russian value of the balanced financial result of enterprises, the ratio to the average Russian value of the occupational level and the ratio to the average Russian value of budget balance (0 is used for negative values in the calculation of the arithmetical mean).

PD is the arithmetical mean of the ratio to the average Russian value of fixed investment per capita, the ratio to the average Russian value of the share of innovatively active organizations, the ratio to the average Russian value of the share of innovative products, and the ratio to the average Russian value of digitization level).

## Declaration of Competing Interest

The data set was developed by Popkova, D.Sc. Economics, Professor, President of the Institute of Scientific Communications. The data set is an original development and intellectual property of the Institute of Scientific Communications. All rights reserved. When using materials from the data set, please provide a link to it: Institute of Scientific Communications (2020). Data Set “Interactive Statistics and Intelligent Analytics of the Balanced State of the Regional Economy of Russia in Terms of Big Data and Blockchain – 2020”. URL: http://archilab.online/en/data/date-set-on-the-regional-economy

## Appendix Supplementary materials

Image, application 1
